# Treatment and practical considerations of diabetic kidney disease

**DOI:** 10.3389/fmed.2023.1264497

**Published:** 2023-12-01

**Authors:** Yara Bilen, Allaa Almoushref, Kenda Alkwatli, Omar Osman, Ali Mehdi, Hanny Sawaf

**Affiliations:** ^1^Cleveland Clinic, Department of Internal Medicine, Cleveland, OH, United States; ^2^Cleveland Clinic, Department of Kidney Medicine, Cleveland, OH, United States; ^3^Cleveland Clinic, Department of Endocrinology, Cleveland, OH, United States

**Keywords:** diabetes, diabetic nephropathy, diabetic kidney disease, chronic kidney disease, SGLT2 inhibitor, finerenone, GLP-1 receptor agonist

## Abstract

Diabetic kidney disease (DKD) is a complication of diabetes that can lead to kidney failure. Over the years, several drugs have been developed to combat this disease. In the early 90s, angiotensin blockade (ACEi and ARBs) was introduced, which revolutionized the treatment of DKD. In recent years, newer drugs such as sodium-glucose co-transporter 2 (SGLT2) inhibitors, glucagon-like peptide-1 (GLP-1) receptor agonists, endothelin antagonists, and mineralocorticoid receptor antagonists (MRA) have shown great promise in reducing albuminuria and protecting the kidneys. These drugs are being used in combination with lifestyle modifications, patient education, and risk factor modification to effectively manage DKD. In this review, we will explore the latest pharmacological options, their efficacy, and their potential to revolutionize the management of this debilitating disease.

## 1 Introduction

Kidney disease is a serious and potentially life-threatening complication of diabetes. It is a progressive condition that can ultimately lead to kidney failure, requiring dialysis or transplantation. Despite advances in monitoring and treating diabetes, it remains the most common cause of end-stage kidney disease. According to the 2022 United States Renal Data System (USRDS), 14.0% of adults have a low estimated glomerular filtration rate (eGFR), albuminuria, or both. The data also showed that the prevalence of diabetes has increased in the most recent years, reaching 9.5% in those without chronic kidney disease (CKD) and 35.6% in those with CKD as of March 2020 (1).

While histology is required to diagnose diabetic nephropathy, most patients with diabetes and kidney disease do not undergo a kidney biopsy and are presumed to have diabetic kidney disease based on clinical parameters. Thus, DKD is a clinical diagnosis made when a patient presents with proteinuria and/or decreased eGFR in the context of long-standing diabetes. In this review, we will not be making the distinction between DKD and proven diabetic nephropathy. However, one has to note that factors other than diabetes might sometimes be implicated (2). This distinction will not be discussed here.

Along with its impact on the kidneys, diabetic kidney disease (DKD) infers an increased risk of cardiovascular disease which is the leading cause of death in people with diabetes and CKD. The management of DKD is multifaceted and it typically involves a combination of lifestyle modifications, patient education, and pharmacotherapy. Over the years, a variety of medications have emerged to combat DKD and its associated complications. These drugs work in different ways to protect the kidneys, mitigate disease progression, and reduce the risk of cardiovascular disease. The first class of medications to revolutionize the management paradigm of DKD are angiotensin-converting enzyme inhibitors (ACEi) and angiotensin receptor blockers (ARB). By blocking the renin-angiotensin aldosterone system (RAAS), these medications can reduce glomerular hypertension, reduce proteinuria, and slow the progression of DKD.

In recent years, newer classes of medications emerged such as sodium-glucose co-transporter 2 (SGLT2) inhibitors, glucagon-like peptide-1 (GLP-1) receptor agonist, endothelin antagonists, and mineralocorticoid receptor antagonists (MRAs) are all showing great promise in the battle against DKD and its cardiovascular morbidity. Despite the availability of these medications, the management of DKD remains a challenge with a significant residual risk remaining even with optimal medical therapy.

## 2 RAAS blockade (ACEi and ABRs)

### 2.1 Introduction

For more than 3 decades, RAAS blockade agents have been the mainstay of treatment in DKD (3). Evidence of their effectiveness dates back to as early as 1983. Further studies have since shown that ACEi and ARBs can slow the deterioration of kidney function (4–6). The main mechanism of action behind this effect is a decrease in glomerular hypertension and hyperfiltration. Other supporting factors include efferent vasodilation, anti-inflammatory and anti-fibrotic effects, decrease in aldosterone and ADH secretion and a decrease in sympathetic activity (6, 7).

### 2.2 Practical considerations

Trials have shown that these agents slow the progression of moderately increased albuminuria to overt proteinuria and even prevent the development of albuminuria in normotensive patients with no albuminuria (8–12). Hence, the maximal tolerated dose of ACEi or ARBs became the antihypertensive regimen of choice for patients with DKD regardless of the disease stage.

However, as with all effective medications, RAAS inhibitors come with a range of side effects. The initiation of ACEi and ARBs may result in elevated levels of serum potassium and creatinine. The reversible drop in eGFR is expected knowing the mechanism of action of these medications and their benefit in reducing single-nephron hyperfiltration. For the majority of patients, these changes are not worrisome. However, in patients with advanced chronic kidney disease who are at risk of volume depletion, acute or chronic kidney injury, and hyperkalemia, it is advisable to monitor kidney function and electrolyte levels approximately 2–4 weeks after starting the medication (13). It is safe to establish a new baseline eGFR as long as the serum creatinine elevation is within 30%, however, if a greater-than-expected increase is observed, the medication should be discontinued until further investigation is completed. As for hyperkalemia, before discontinuing the drug or reducing the dose, patients’ diet should be reviewed, limiting very high potassium content, keeping in mind that potassium-rich foods are important for the reduction of cardiovascular events (14). Physicians should make sure patients are not on any potassium supplements while promoting healthy bowel movements (15). Potassium binders such as patiromer can also be used to significantly reduce serum potassium, helping to achieve the desired dose of RAAS inhibition while limiting hyperkalemia (16). Several factors could lead to hyperkalemia and elevation in creatinine while on ACEi or ARBs including hypovolemia, concomitant use of NSAIDs, and bilateral RAAS inhibition. Thus, potassium levels >5.6 mmol/L refractory to the above-mentioned adjustments within the first 2 months of treatment prompt withdrawal of the medication or dose change (13). It is considered safe to restart or uptitrate the ACEi or ARB dose after the initial insult has resolved and labs have normalized.

Cough and angioedema are also important barriers seen mainly with ACEi. Switching patients who exhibit these side effects to an ARB has been subject to debate. According to the American Heart Association, it is reasonable to switch to an ARB in patients developing ACEi-provoked cough or angioedema. It is preferred that patients undergo a 6-week washout period between stopping the ACEi and starting the ARB (Class 1 recommendation with level A evidence) (15).

It is important to note that women of childbearing age should be on contraception while on ACEi or ARBs and guidelines recommend stopping these agents if pregnancy were to occur due to their teratogenic effects (17, 18).

Holding RAAS inhibitors below a certain GFR cutoff has been subject to debate. In the recent STOP ACEi trial, patients with advanced and progressive chronic kidney disease (eGFR, <30 mL per minute per 1.73 m^2^ of body-surface area) were randomized into either to discontinue or continue the therapy with RAAS inhibition. This multi-centered, open-label study showed no significant difference in the long-term rate of eGFR decline between the two arms. It is thus recommended not to discontinue RAAS inhibition unless treatment is complicated by side effects such as refractory hyperkalemia. This is of particular importance in patients with additional indications for RAAS inhibition (19) (Table 1).

**Table 1:** Practical consideration for RAAS inhibitors.

Medication	Practical consideration
RAAS inhibitors	Hyperkalemia and decline in GFR: Monitor with lab draws. Consider a low-potassium diet and potassium binders before discontinuing the drug. Teratogenicity: Rule out pregnancy and consider contraception before drug initiation. Angioedema and cough with ACEi: Stop medication and consider switching to ARB. Combination therapy: Do not combine ACEi with ARB therapy

### 2.3 Evidence

Several landmark trials have revolutionized our practice regarding the utility of RAAS inhibition. The CSG Captopril in 1993 showed the benefit of an ACE inhibition on the progression of kidney disease in patients with insulin-dependent diabetes mellitus (20). Subsequently, large randomized clinical trials, have further supported the importance of RAAS blockade in preventing kidney failure in DKD, including the Reduction of Endpoints in non-insulin-dependent diabetes mellitus with the Angiotensin II Antagonist Losartan (RENAAL) and Irbesartan in Diabetic Nephropathy Trial (IDNT). In both trials, ARBs showed a reduction in the primary endpoint of doubling creatinine, ESKD, and death compared to conventional hypertension therapy (5, 21). In the RENAAL trial, though the losartan arm did not have any mortality benefit, it did show a 25 and 28% risk reduction for creatinine doubling and ESKD, respectively (5). Finally, the Ongoing Telmisartan Alone and in combination with Ramipril Global Endpoint Trial (ONTARGET) and the Veterans Affairs nephropathy in Diabetes (VA Nephron D) study showed that the combination of ACE inhibition and ARB together lead to more adverse events with little additional benefit. While the practice of dual RAAS blockade was common at some point, given the above, this practice is not recommended (22, 23).

## 3 Sodium-glucose transport protein 2 inhibitors

### 3.1 Introduction

**Figure 1:**
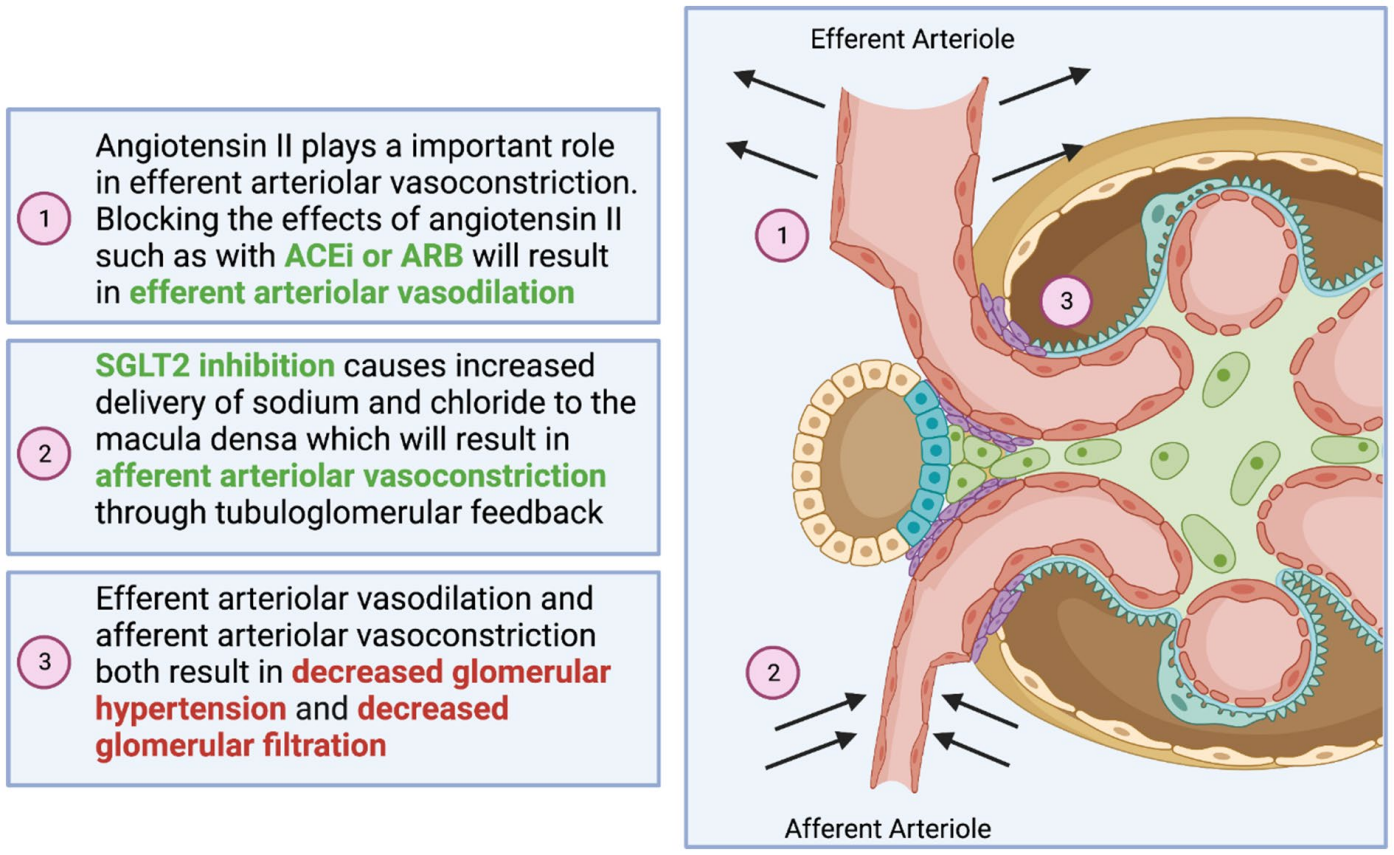
Visual representation of the synergistic effect of angiotensin-converting enzyme inhibitors (ACEi), angiotensin receptor blockers (ARB), and SGLT2 inhibitors.

Sodium-glucose co-transporter 2 inhibitors were introduced as glucose-lowering agents in patients with type 2 diabetes by enhancing glucosuria (24). In 2008, the US FDA mandated that any agent approved for glucose lowering in type 2 diabetes was required to undergo a cardiovascular safety trial (25). It was found that not only were SGLT2 inhibitors safe, but also they were highly effective in terms of reducing cardiovascular risks (26). Furthermore, the major secondary outcomes in these trials showed improvement in kidney outcomes (27, 28). The use of dapagliflozin also showed mortality benefit in the DAPA-CKD trial revolutionizing the treatment of patients with type 2 diabetes and chronic kidney disease (CKD) (29). Note that, this class of medications has a modest effect on lowering HgbA1c, systolic blood pressure, and weight loss through their glycosuria and metabolic effects (28, 30). While the latter are all favorable effects, however, they are not the primary mechanisms responsible for the substantial cardiovascular and kidney protection seen in landmark trials. The most plausible mechanism of SGLT2 inhibitors resulting in kidney protection is believed to be the restoration of the tubuloglomerular feedback. In patients with type 2 diabetes, increased glomerular hypertension and glomerular hyperfiltration are the main maladaptive mechanisms involved in the pathogenesis of DKD (31). The action of SGLT2 inhibitors in the proximal convoluted tubule increases sodium delivery to the macula densa leading to vasoconstriction of the afferent arterioles and ultimately lowering the intraglomerular pressure (32). There are other non-hemodynamic effects of SGLT2 inhibitors which include anti-inflammatory and antifibrotic pathways (33). Other studies suggested that they can cause a reduction in oxidative stress (34) and increase erythropoietin production (35). Furthermore, it has been hypothesized that SGLT2 inhibitors induce mild ketosis, which has a cardiovascular benefit by providing beta-hydroxybutyrate, a preferred myocardial fuel leading to increased ATP production and enhancing cardiac contractility (36). All these mechanisms among others are still under active investigation, but what is clear is that the great benefit of SGLT2 inhibitors in heart disease contributes to their favorable effects on the kidneys by slowing down the vicious cardiorenal circle in diabetes (37) (Figure 1).

### 3.2 Practical consideration

According to the American Diabetes Association (ADA) and Kidney Disease Improving Global Outcomes (KDIGO) guidelines, SGLT2 inhibitors are now recommended first-line agents for patients with type 2 diabetes, CKD, and eGFR ≥20 mL/min/1.73 m^2^ with urinary albumin >200 mg/g creatinine (Grade A recommendation) (38, 39). In 2023, the ADA updated the recommendation for standards of care in patients with CKD and diabetes to recommend the use of SGLT2 inhibitors even with urinary albumin ranging from normal to 200 mg/g creatinine (Grade B recommendation) following the EMPA-KIDNEY trial (40).

The use of SGLT2 inhibitors in this lower level of eGFR is mainly for renal and cardiovascular protection, regardless of glycemic targets. It is essential to know that with the initiation of an SGLT2 inhibitor, it is expected to see an early reversible decline in eGFR (5–8 mL/min/1.7m^2^) in the first 2 weeks of initiation, which is explained by the hemodynamic effect of this medication in reducing glomerular hyperfiltration (41). In elderly patients with advanced CKD, it is reasonable to check kidney function 2–4 weeks after initiation to establish a new baseline eGFR. A more-than-expected decline in eGFR should prompt the discontinuation of SGLT2 inhibitors until further evaluation is completed.

It is important to recognize the potential side effects of this drug class. One concerning side effect is genital mycotic infections which can occur in 6% of participants assigned to an SGLT2 inhibition (42). This risk is reduced by self-care practices of daily hygiene, including but not limited to rinsing the genital area with water after every void and before going to bed to minimize the risk of urinary tract infection (UTI) and Fournier’s gangarene (43). Another side effect to keep in mind is the increased risk of diabetic ketoacidosis (DKA) as well as euglycemic DKA, which is generally uncommon. Patients should be educated for early recognition of this side effect. Regular serum or urine ketone monitoring may be considered in high-risk populations such as those with a prior history of DKA. Treatment with SGLT2 inhibitors should be approached with caution if not avoided altogether in individuals with a history of DKA. The development of DKA is the main reason why the use of SGLT2 inhibitors has not been approved for patients with type 1 diabetes in the United States, although there are reports of safe use from European registries (44, 45).

Even though SGLT2 inhibitors do not cause hypoglycemia themselves, there is an improvement in glycemic control in some patients. Therefore, dose adjustment of insulin and insulin secretagogues such as sulfonylureas may be needed (46). This is more notable in patients with preserved GFR above 45 mL/min. However, maintaining at least a low insulin dose for patients already on it is also vital to reducing this DKA risk.

Given the potential risk of hypovolemia, hypoglycemia, and DKA, a “sick day protocol” can be implemented where pausing SGLT2 inhibitor treatment is considered during acute illness, vomiting, diarrhea, and inability to eat or drink (4). For similar reasons, decreasing the diuretic dose before SGLT2 inhibition use may be considered for patients at risk of hypovolemia, particularly elderly patients. Patients should be educated about volume depletion and hypotension signs and symptoms. In general, SGLT2 inhibitors can be restarted 24–48 h following recovery (47).

The risk of lower limb amputations was of concern noted in the Canvas program, however, this risk was not noted in other studies (48). However, we do refrain from starting SGLT2i in patients with open wounds.

Non-steroidal anti-inflammatory drugs (NSAIDs) can cause medullary hypoxic injury and vasoconstriction of the afferent renal arteriole, reducing the kidney’s ability to regulate glomerular blood flow (49). Therefore, the concomitant use of SGLT2 inhibitors with NSAIDs should be avoided.

Finally, it is important to mention that in all landmark trials involving kidney outcomes using SGLT2 inhibitors, patients were on a maximally tolerated RAAS inhibition medication (in the form of ACEi or ARB) before starting SGLT2 inhibitors. Therefore, optimizing RAAS inhibition is important prior to SGLT2 inhibitor initiation (Table 2).

**Table 2:** Practical consideration for SGLT2i.

Medication	Practical consideration
SGLT2 inhibitors	Do not initiale with eGFR < 20 mL/min/1.73 m^2^: These patients were excluded from the trials. If already on one, continue until dialysis initiation. Decline of GFR upon initiation: Monitor with blood work at 2–4 weeks post initiation and stop medication if a greater-than-expected decline is noted. Mycotic infections: Daily hygiene and self-care. DKA: Education and regular serum and urine ketones for high-risk patients. Hypoglycemia: Monitor concomitant insulin and/or insulin secretagogues dose. Hypovolemia: Monitor concomitant diuretic dose. Implement “sick day protocol” in the setting of acute illness causing poor PO intake, nausea, vomiting, and/or diarrhea.

### 3.3 Evidence

Since 2017, several landmark trials related to SGLT2 inhibition have been conducted. The CREDENCE trial was the first landmark placebo control trial for an SGLT2 inhibitor where the primary outcome being evaluated were the renal outcomes. Canagliflozin in patients with type 2 diabetes and kidney disease was shown to result in a 30% relative risk reduction of kidney disease when compared to placebo (50). The DAPA–CKD trial in 2020 was the first kidney disease outcome trial to include a substantial proportion of participants with and without type 2 diabetes. The trial included participants with eGFR 25–75 mL/min/1.73m^2^ and albuminuria of 200–5,000 mg/g (29). The primary kidney outcome (sustained ≥50% eGFR decline, kidney failure, or cardiovascular or renal death) was reduced by 39% in the dapagliflozin arm compared to the placebo, with similar effects in patients with and without type 2 diabetes. Dapagliflozin also reduced all-cause mortality by 31% in patients with and without type 2 diabetes. Dapagliflozin was the first drug in the nephrology world that was given fast-track, breakthrough, and priority approval by the US Food and Drug Administration (FDA) in April 2021 due to its profound benefit on kidney outcomes and all-cause mortality. The most recent landmark trial, the EMPA-KIDNEY trial, included adults with or without diabetes and aimed to determine the benefit of empagliflozin in patients with low eGFR (20 mL/min/1.7m^2^) with and without albuminuria. The primary outcome of kidney disease progression or death from cardiovascular causes was reduced by 28% in the empagliflozin group compared to the placebo group (40). This suggests that the effect of SGLT2 inhibition can be seen across the entire cohort of CKD patients, however, the benefit was more pronounced in patients with proteinuria. This trial was also stopped early because of clear positive efficacy. As a result of this trial the European Medicines Agency (EMA), and most recently the FDA approved empagliflozine for the treatment of adult patients with chronic kidney disease with or without diabetes.

## 4 Glucagon-like peptide-1 receptor agonists

### 4.1 Introduction

Glucagon-like peptide-1 receptor agonists have been used to treat type 2 diabetes since 2005 (51). GLP-1 increases insulin sensitivity and secretion from pancreatic beta cells and increases its proliferation. It also slows gastric emptying and promotes satiety (52). Exenatide was the first GLP-1 receptor agonist on the market and was manufactured from a salivary hormone (exendin 4) (53). Since that time, multiple medications of this drug class have been released, including lixisenatide, liraglutide, dulaglutide, and semaglutide.

**Figure 2:**
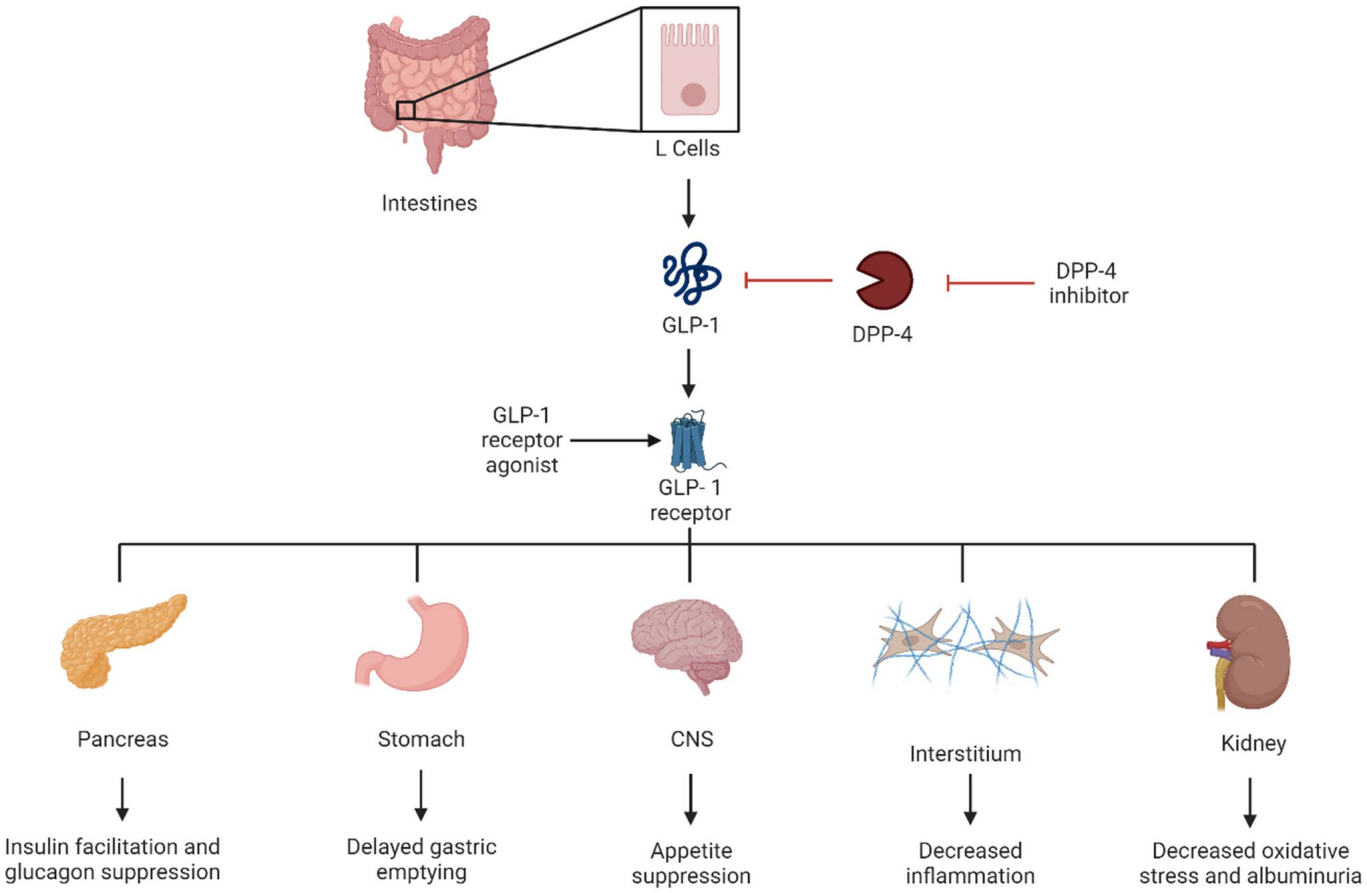
Visual representation of the effect of GLP-1.

Tirazepatide is a new medication that, in addition to being a GLP-1 receptor agonist, also activates the receptors of glucose-dependent insulin tropic polypeptide (GIP) which improves insulin sensitivity and secretion in a way similar to GLP-1 (54). Kidney protection is one of the numerous beneficial effects of some GLP-1 receptor agonists. Suggested mechanisms include blocking angiotensin II inflammatory effects and oxidative stress and decreasing glomeruli hyperfiltration in animal models (4). Other favorable benefits include weight loss, cardiovascular benefits, and cerebrovascular benefits (55), which make this drug class the first line of recommended anti-hyperglycemic medications by the ADA, particularly in patients with overweight or obesity, and in patients who have non-proteinuric kidney disease (39) (Figure 2).

### 4.2 Practical considerations

It is important that clinicians familiarize themselves with the different medications in this class, since not all of them share the same degree of benefits. When looking at their utilities from a kidney standpoint, only liraglutide, dulaglutide, and subcutaneous semaglutide have demonstrated kidney benefits and effects on major cardiovascular events (56–59). Importantly, no dosage adjustment is needed in patients with kidney disease. This is contrary to older GLP-1 receptor agonists, such as exenatide and lixisenatide, which are not recommended in eGFR < 45 and eGFR < 15, respectively. Tirazepatide has demonstrated the maximum benefits on weight loss among all weight loss-promoting medications (up to 20.9% mean percentage change in weight at week 72 when the maximum dosage of tirazepatide is used vs. −14.9% at week 68 with the maximum dosage of semaglutide and −8% at 56 weeks with liraglutide 3 mg) (60); its benefits on renal and cardiovascular disease are still under investigation at the time of this review.

When patients on insulin treatment begin taking a GLP-1 receptor agonist, it is crucial to monitor their insulin dosage closely. Increasing the dose of the GLP-1 receptor agonist may require a decrease in insulin dosage and adjustment or discontinuation of insulin secretagogues if hypoglycemia is a concern.

Glucagon-like peptide-1 receptor agonists can be used as a combination therapy with insulin and other oral medications except for other incretin therapies such as DPP-4 inhibitors; this combination is unlikely to provide additional benefits on glycemic targets.

Clinicians must counsel patients on potential side effects, particularly gastrointestinal effects which tend to be temporary and can be mitigated by starting with a low dose and up-titration every 2–4 weeks. Providing patients with dietary consultation, including eating small meals with low-fat content and avoiding spicy food, might help reduce their symptoms. Patients with underlying gastroparesis might benefit from a slower titration. In patients with severe forms of gastroparesis, GLP-1 receptor agonist should be avoided.

Another consideration is to evaluate patients for gallbladder disease before initiating the medication in patients with symptoms suggestive of cholelithiasis. Pancreatitis has been reported in association with GLP-1 receptor agonist use; however, causality has not been established. If patients develop symptoms suggestive of pancreatitis, the medication should be discontinued.

Medications of this class are contraindicated in patients with a personal or family history of medullary thyroid cancer (c-cell tumors). Although the risk was only seen in animal models, human prevalence is not determined. None of the other more common thyroid cancers have been associated with an increased risk. Clinicians should reassure patients with a personal or family history of non-c-cell thyroid tumors.

Glucagon-like peptide-1 receptor agonists’ effect on retinopathy has been debated for the past years. Studies using data from cardiovascular outcome trials suggested an association between retinopathy progression and GLP-1 receptor agonist use. However, one could argue that retinopathy can worsen with any treatment that causes rapid hemoglobin A1c reduction. Furthermore, an analysis of the FDA Adverse Event Reporting System showed no evidence that GLP-1 receptor agonists are associated with adverse effects related to retinopathy progression. More studies showed no effect of GLP-1 receptor agonists on angiogenesis and no association between GLP-1 agonist exposure and severe diabetic retinopathy. In clinical practice, we suggest close follow-up in patients with a history of moderate to severe retinopathy, particularly in patients who experience a rapid reduction of hemoglobin A1c levels with GLP-1 receptor agonist treatment (Table 3).

**Table 3:** Practical consideration for GLP-1 receptor agonists.

Medication	Practical consideration
GLP-1 receptor agonists	Hypoglycemia: Monitor required insulin and/or insulin secretagogue dose. Gastrointestinal side effects: Initiating slow dose and up titrate as tolerated. Recommend dietary changes. Gallbladder disease: Evaluate if symptoms of cholelithiasis are of concern. Pancreatitis: Stop medication. Medullary thyroid cancer: Medication counter-indicated in patients with history or family history of MTC. Retinopathy: Close follow-up in patients with moderate to severe retinopathy, particularly those who experience a rapid reduction of hemoglobin A1C levels with GLP-1 receptor agonist therapy.

### 4.3 Evidence

An analysis of data presented in the ELIXA trial, which compared lixisenatide to placebo, showed that lixisenatide reduced the urinary albumin-to-creatinine ratio (UACR) and it was only statistically significant in participants with stage A3 albuminuria (39.2%; 95% CI = −68.5 to −9.8) (61, 62). A *post hoc* analysis of EXSCEL trial data compared exenatide to placebo which showed decreased onset of severe albuminuria in the exenatide group, but the difference was not statistically significant (34.5%; 95% CI = −54.4 to −6.0) (63, 64).

The LEADER trial, which studied liraglutide vs. placebo, showed renal benefit in patients with type 2 diabetes as lower rates of nephropathy events were seen in the liraglutide group (13%) compared to placebo group (14.9%) (57). SUSTAIN-6 studied semaglutide vs. placebo with nephropathy as a secondary outcome; new or worsening nephropathy occurred in 3.8% in the semaglutide group vs. 6.1% in the placebo (56).

The AWARD-7 trial compared dulaglutide to insulin glargine in patients with moderate to severe kidney disease and type 2 diabetes; a decrease in UACR was seen in patients receiving dulaglutide 1.5 mg weekly (59). The REWIND trial studied dulaglutide vs. placebo, showing less new-onset stage A3 albuminuria in the dulaglutide group (58). Of note, kidney disease outcomes were secondary in all mentioned trials. However, there is an ongoing dedicated kidney outcomes trial designed to determine the kidney-protective effects of semaglutide in participants with CKD and T2D (the FLOW trial). This study was just stopped early due to an interim analysis suggesting a very high likelihood of study success. Full results are now expected in early 2024.

Looking specifically at the GLP-GIP agonist tirzepatide, a *post hoc* analysis of data from the tirzepatide trial (SURPASS-4) showed a slower rate of eGFR decline (−1·4 mL/min per 1·73 m^2^ per year) in the tirzepatide group compared to the insulin glargine group (−3·6 mL/min per 1·73 m^2^ per year); UACR increased from baseline in the insulin-treated group, whereas there was no significant increase observed in the group receiving tirzepatide (65).

A meta-analysis published by Kristensen et al. (66) included all the studies mentioned above (except the AWARD-7 trial) and showed a 17% reduction in a composite kidney outcome (development of stage A3 albuminuria, worsening kidney function, end-stage kidney disease, and kidney-related death) in a patient treated with GLP-1 receptor agonists.

## 5 Mineralocorticoid receptor antagonists

### 5.1 Introduction

**Figure 3:**
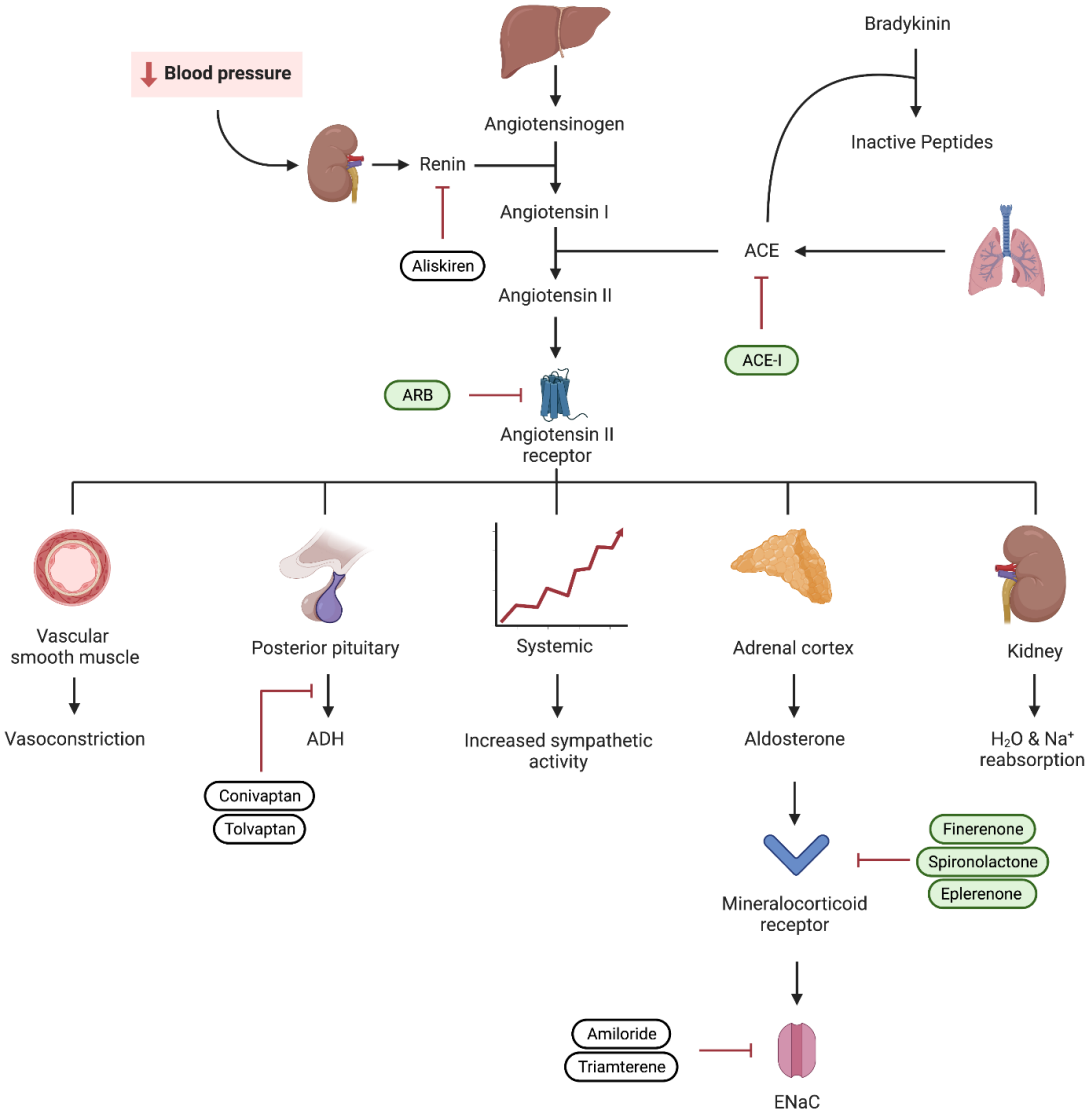
Visual representation of the renin–angiotensin–aldosterone system (RAAS) and the mechanism of ACEi, angiotensin receptor blockers (ARB), and mineralocorticoid receptor blockers. Medications used in diabetic kidney disease are depicted in green.

Multiple studies have highlighted the potential benefit of MRAs in managing hypertension and reducing proteinuria. In addition to blocking the epithelial sodium channel in the principal cell and decreasing sodium reabsorption, MRAs have been demonstrated to decrease inflammation, oxidative stress, and scarring (67, 68). While steroidal MRAs like spironolactone and eplerenone have been available for some time, their use is limited by their side effects (69). The newest addition to our repertoire is non-steroidal MRAs (ns-MRAs), with the prototype, finerenone. These newer medications have demonstrated protective cardiorenal effects with a more favorable risk-to-benefit ratio compared to their steroidal counterparts (70) (Figure 3).

### 5.2 Practical consideration

Prior to initiating treatment with MRAs, it is essential to consider several factors. Firstly, the patient should already be receiving maximal tolerated doses of RAAS and SGLT2 inhibition.

Secondly, when faced with uncontrolled hypertension and hyperaldosteronism, it is preferable to add a steroidal MRA such as spironolactone and eplerenone rather than finerenone due to the limited blood pressure effect of the latter. However, the use of spironolactone is limited by hyperkalemia and gynecomastia. It has been shown that the addition of a potassium binder in conjunction with spironolactone decreased the rate of drug discontinuation, particularly in advanced CKD (71). Eplerenone has less binding affinity at androgen receptors and is a viable alternative in patients who experience enlargement of breast tissue with spironolactone (72).

The KDIGO consensus and the most recent 2023 ADA guideline currently support the addition of ns-MRAs to prevent the progression of DKD in the setting of a normal potassium and an eGFR ≥ 25 mL/min/1.73 m^2^ and albumin creatinine ratio ≥ 30 mg/g, despite current standards of care.

The trials evaluating the effectiveness of ns-MRA in patients with DKD were conducted before the benefits of SGLT2 inhibitors were well established. In fact, only a small percentage of patients (4.6 and 8.4% in FIDELIO and FIGARO, respectively) were on SGLT2 inhibitors during these trials. Currently, it is recommended that providers initiate and prioritize maximally tolerated RAAS and SGLT2 inhibition as the standard of care before considering additional treatment like finerenone, despite limited data on the efficacy of ns-MRA in patients receiving both RAAS and SGLT2 inhibition.

Finerenone should be started at 20 mg in patients with eGFR ≥ 60 mL/min/1.73 m^2^ and 10 mg in patients with eGFR 25–60 mL/min/1.73 m^2^ then up titrated to 20 mg as tolerated.

Despite being used to address concerns around hyperkalemia, it is worth noting that ns-MRAs can still result in the elevation of potassium levels in a notable percentage of patients, with rates ranging from 11 to 16% based on data from the FIDELIO and FIGARO clinical trials (70). The concomitant use of SGLT2 inhibitors with their potential kaliuretic effect might help mitigate the hyperkalemia challenge.

During treatment, potassium levels should be followed regularly. It is safe to increase the dose from 10 to 20 mg for patients with potassium <4.8 mmoL/L and recheck levels in 4 weeks post-dose up-titration. Finererone should be withheld if potassium increases to >5.5 mmoL/L. If hyperkalemia was the limiting factor preventing dose up-titration, it is recommended to resume the medication at a lower dose after the achievement of normokalaemia on follow-up labs.

It is also worth mentioning that, although these medications are not primarily used as antihypertensives as mentioned above, they still have considerable impact on lowering blood pressure. Placebo-adjusted change in 24-h ambulatory blood pressure measurements at day 90 was −8.3 mmHg (95% confidence interval [CI], −16.6 to 0.1) for finerenone 10 mg (*n* = 27), −11.2 mmHg (95% CI, −18.8 to −3.6) for finerenone 15 mg (*n* = 34), and − 9.9 mmHg (95% CI, −17.7 to −2.0) for finerenone 20 mg (*n* = 31) (73, 74).

Lastly, it should be noted that concomitant use of both steroidal and ns-MRAs is not recommended (Table 4).

**Table 4:** Practical consideration for MRA.

Medication	Practical consideration
MRAs	Hyperkalemia: Monitor with blood work, try low potassium diet and potassium binders before discontinuing the drug. Gynecomastia with spironolactone: Switch to eplerenone. Hypertension: Unlike spironolactone and eplerenone, finerinone is not primarily used as an antihypertensive agent. Teratogenicity: Not recommended during pregnancy.

### 5.3 Evidence

Spironolactone has demonstrated promising efficacy in managing DKD, particularly in reducing proteinuria and slowing the progression of kidney damage. Several reviews including a Chocrane systematic review confirmed the additional benefit of steroidal MRAs for kidney and cardiac protection when used with an ACEior ARB (75, 76). Adding spironolactone to standard treatment decreased urinary albumin/protein excretion and increased reduction in urinary albumin to creatinine ratio (77). A study by Mehdi et al. (78) showed a reduction of the urinary albumin to creatinine ratio by 34.0% (95% confidence interval [CI]: −51.0 to −11.2%, *p* = 0.007) in the spironolactone group and by 16.8% (95% CI: −37.3 to +10.5%, *p* = 0.20) in the losartan group.

Multiple phase II randomized clinical trials investigated the efficacy and safety of finerenone. Fewer side effects were seen with the ns-MRA as highlighted in the Mineralocorticoid Receptor Agonist Tolerability Trial (ARTS) showing significantly less hyperkalemia compared to spironolactone (79). A dose-dependent decrease in albuminuria was seen in the subsequent ARTS-DN trial further paving the path for further investigations (80).

In more recent years, phase III trials namely the finerenone in reducing kidney failure and decreasing progression of diabetic kidney disease (FIDELIO-DKD) and the finerenone in reducing cardiovascular mortality and morbidity in diabetic kidney disease (FIGARO-DKD) provided the biggest evidence of cardiorenal protection. The composite kidney outcome occurred in 5.5% of patients receiving finerenone and 7.1% receiving placebo (70). Once again, finerenone proved to reduce the risk of cardiovascular and kidney outcomes in diabetics on maximal dose ACE inhibitor or ARB, with lower rates of hyperkalemia compared to placebo across stages of kidney disease (70).

## 6 Endothelin antagonists

### 6.1 Introduction

Endothelins were first discovered in 1985 with the first endothelin aptly named endothelin 1. Endothelin 1 has simultaneously been implicated in inflammation, vasoconstriction, and mesangial proliferative effects mediated by endothelin receptor A. These derangements are implicated in the underlying pathophysiology, both initial insult and ongoing injury/progression of DKD, hypertension, and glomerulonephritis (81). There is also evidence for overexpression of endothelin receptors in diabetics. Antagonism of the endothelin receptor was shown to aid with microcirculation in animal models, however, similar effects have yet to be shown in human trials (82). Endothelin A receptor blockade has multiple effects including a reduction in glomerular vasodilation which can also alter permeability for proteins including albumin leading to a lower tubular load of protein excretion (83). Though sparsentan has been granted accelerated FDA approval for the treatment of IgA nephropathy in adults, there are currently no medications approved for treatment of DKD in this class.

### 6.2 Practical consideration

Volume overload and heart failure exacerbations remain a concern when for treatments using endothelin receptor antagonists. Combining endothelin receptor antagonists with SGLT2 inhibitors may reduce fluid retention similar to thiazides or loop diuretics (84). This can potentially be explained by a synergistic effect between medication classes and their effect (85). This hypothesis is being evaluated in the ZENITH trial. This trial randomized groups (CKD patients with and without diabetes) to receive zibotentan combined with dapagliflozin. Recruitment for this study has been completed at the time of writing but results are pending (86).

When prescribing endothelin antagonist, other effects should be considered beyond the medication’s effect on volume overload. First, it is key to exclude pregnancy before starting treatment and 1 month after stopping the medication. This recommendation is mainly due to the teratogenic effect of these drugs seen in previous endothelin receptor agonist’s studies for pulmonary arterial hypertension (87). It is also recommended to monitor for hemoglobin in the first 3 months of drug initiation, as a drop in hemoglobin was noted during that time. Hemoglobin was noted to stabilize after that period (84). Finally, current FDA guidelines recommend monthly monitoring of liver enzymes since elevation and liver injury were reported in several ERAs (88, 89). Discontinuation of the medication is advised if liver enzymes increase more than five times the upper limit of normal, or if bilirubin increases more than twice the upper limit of normal, or if clinical signs of liver toxicity or failure are seen (although no serious liver injury was noted in ASCEND or SONAR trials) (89) (Table 5).

**Table 5:** Practical considerations for endothelin receptor antagonists.

Medication	Practical consideration
Endothelin receptor antagonists	Volume overload: Use diuretics to optimize volume status. Liver injury: Monitor liver enzymes. Anemia: Monitor for hemoglobin in the first three months of drug initiation. Teratogenicity: Rule out pregnancy and start contraception before drug initiation.

### 6.3 Evidence

The ASCEND trial evaluated kidney composite outcomes in patients receiving either avosentan or placebo. Due to safety concerns relating to volume overload and heart failure exacerbations, the trial was terminated early. Despite having a statistically significant reduction in albuminuria in patients on avosentan, there were no differences in kidney composite outcomes (90). The SONAR trial evaluated whether endothelin antagonism could be of benefit in certain groups of patients with diabetic kidney disease. The study followed a pragmatic trial design in which patients with diabetic kidney disease and proteinuria despite maximal tolerated RAAS blockade were treated with atrasentan during an enrichment period. Patients who did not develop significant volume retention were then randomized to receive atrasentan vs. placebo. Results showed improved kidney outcomes in patients who tolerated the endothelin antagonist. 6.0% of patients in the atrasentan group and 7.9% in the placebo group had a primary composite renal endpoint event including doubling of serum creatinine (sustained for ≥30 days) or end-stage kidney disease (eGFR <15 mL/min per 1.73 m^2^ sustained for ≥90 days, chronic dialysis for ≥90 days, kidney transplantation, or death from kidney failure) (91). Patients in the treatment arm had fewer primary composite renal endpoint events (6% as opposed to 7.9% in the placebo group) which included the doubling of serum creatinine (sustained for ≥30 days) or end-stage kidney disease (eGFR <15 mL/min per 1.73 m^2^ sustained for ≥90 days, chronic dialysis for ≥90 days, kidney transplantation, or death from kidney failure) (91).

## 7 Promising therapeutic options

New alternative treatment options encompass antifibrotic interventions utilizing pirferidone or pentoxifylline (92), Nox1/4 inhibition (93, 94), and chemokine cytokine inhibition (95). Other pharmacological options targeting several inflammatory pathways have been the subject of interest. Most recently baricitinib, a JAK1/2 inhibitor, was shown to decrease albuminuria in patients with type two diabetes and DKD (96). Bardoxolone methyl has also been studied since it activates the Keap1/Nrf2 system which plays an important role in defense responses against oxidative stress (97). Other herbal supplements with antioxidant properties have also been investigated such as silymarin, but more research is needed before adding these agents to our growing list of management options (98). Preliminary findings suggest that DDP-4 inhibitors such as saxagliptin and linagliptin, may offer potential advantages for patients with DKD (99). Overall research is moving toward a more personalized approach based on the patient’s genetic and biomarker profile as the future of DKD management (100).

## 8 Conclusion/summary

Several new agents have made a significant impact in the field of DKD with clear protective advantages not only in terms of kidney disease progression but also in cardiovascular risk mitigation. While RAAS inhibitors continue to be essential for managing these patients, we now have the option of offering additional medications that can complement the benefit of blocking RAAS including SGLT2 inhibitors, GLP-1 receptor agonists, and MRA.

In summary, borrowing the terminology from our heart failure colleagues, the guideline-directed medical therapy for DKD is here and for the time being includes ACEi or ARB, SGLT2 inhibitors, GLP-1 receptor agonist, and an MRA. The expansion of therapeutic options has marked the beginning of a new era in DKD management where we can hopefully be more impactful in the care of these patients (Table 6).

**Table 6:** Summary of medication side effects and clinical considerations.

Medication	Practical consideration
RAAS inhibition	Hyperkalemia and decline in GFR: Monitor with lab draws. Consider a low potassium diet and potassium binders before discontinuing the drug. Teratogenicity: Rule out pregnancy and consider contraception before drug initiation. Angioedema and cough with ACEi: Stop medication and consider switching to ARB. Combination therapy: Do not combine ACEi with ARB therapy.
SGLT2 inhibitors	Do not initialize with eGFR < 20 mL/min/1.73 m^2^: These patients were excluded from the trials. If already on one, continue until dialysis initiation. Decline of GFR upon initiation: Monitor with blood work at 2–4 weeks post initiation and stop the medication if a greater-than expected decline is noted. Mycotic infections: Daily hygiene and self-care. DKA: Education and regular serum and urine ketones for high-risk patients. Hypoglycemia: Monitor concomitant insulin and/or insulin secretagogues dose. Hypovolemia: Monitor concomitant diuretic dose. Implement “sick day protocol” in the setting of acute illness causing poor PO intake, nausea, vomiting, and/or diarrhea.
GLP-1 receptor agonist	Hypoglycemia: Monitor required insulin and/or insulin secretagogue dose. Gastrointestinal side effects: Initiating slow dose and up titrate as tolerated. Recommend dietary changes. Gallbladder disease: Evaluate if symptoms of cholelithiasis are of concern. Pancreatitis: Stop medication. Medullary thyroid cancer: Medication counter-indicated in patients with a history or family history of MTC. Retinopathy: Close follow-up in patients with moderate to severe retinopathy, particularly those who experience a rapid reduction of hemoglobin A1C levels with GLP-1 receptor agonist therapy.
MRAs	Hyperkalemia: Monitor with RFP, try a low potassium diet and potassium binders before discontinuing the drug. Gynecomastia with spironolactone: Switch to eplerenone. Hypertension: Unlike spironolactone and eplerenone, finerinone is not primarily used as an antihypertensive agent. Teratogenicity: Not recommended during pregnancy.
Endothelin receptor antagonists	Volume overload: Use diuretics to optimize volume status. Liver injury: Monitor liver enzymes. Teratogenicity: Rule out pregnancy and start contraception before drug initiation. Anemia: Monitor for hemoglobin in the first three months of drug initiation.

## Author contributions

YB: Writing – original draft. AA: Writing – original draft. KA: Writing – original draft. OO: Writing – original draft. AM: Conceptualization, Supervision, Writing – review & editing. HS: Conceptualization, Supervision, Writing – review & editing.
